# Supplementary material for: Psychological stress and associated factors among municipal solid waste collectors in Hanoi, Vietnam: A cross-sectional study
Source: PLoS One. 2021 Jul 12;16(7):e0254355. doi: 10.1371/journal.pone.0254355 (PMC8274853; doi:10.1371/journal.pone.0254355)
Supplement: S1 Data — (DOCX) [file pone.0254355.s001.docx]

SUPPORTING DOCUMENT: RESEARCH QUESTIONNAIRES IN ENGLISH AND VIETNAMESE

THE SITUATION OF STRESS AND RELATED FACTORS AMONG HANOI URBAN ENVIRONMENT WORKERS IN 2017

Investigator: ………………………………………………………….......

Interview date: ………………………………………………………….

Address: …….……………………… Phone: …………………..................

**INTRODUCTION**

*Dear Mr/ Ms, my name is…………….,* *from the Hanoi University of Public Health.* *Currently, I am working on a study to understand the relationship between personal factors and environmental factors, working conditions and psychological stress among waste collectors.* *This study aims to provide more information for the identification and assessment of psychological stress that may be encountered in waste collectors in Hanoi. The results of this study will give solutions for prevention of psychological stress. All information you provide will be kept confidential and used only for the purpose of this research. We hope you agree to participate in the study. During the interview, you have the right to refuse to answer questions that you do not want to answer or stop interviewing at any time*

*After the interview, if you have any questions related to this research, please contact us by phone number:*

Our interview will last for about 20 minutes

**Do you agree to participate in this interview?**

[ ] Agree [ ] refuse

***Thank you for your cooperation!***

**1. PERSONAL INFORAMTION**

1.1. Year of birth:…………….

1.2 Gender: 1. Male 2. Female 3. Other

1.3. Education attainment:

1. Elementary 2. Secondary 3. High school 4. Vocational 5. College and higher

1.4. Marriage status:

1. Single 2. Married and live with spouse 3. Divorced/separated 4. Other

1.5. Number of children under 5 years old:

1. No child 2. Children >5 3. 1-2 children < 5 4. > 2 children > 5

1.6. How long have you worked as a waste collector??........... years……. months

1.7. What is the average number of hours that you work every day? …………. hours

1.8. During the last 3 months, what shift do you work?

1. Morning (5a.m-12a.m) 2. Afternoon (1p.m-8plm) 3. Night (6p.m-2a.m) 4. Frequently change

1.8. How much is your average monthly income? ……………. Million VND

**2. WORKING CONDITIONS**

How is your exposure to the following occupational hazards at work?

| **No** | **Hazards** | **Level of exposure** | | |
| --- | --- | --- | --- | --- |
|  |  | No | Seasonal | Frequent |
| 2.1 | Heat | 1 | 2 | 3 |
| 2.2 | Sunlight | 1 | 2 | 3 |
| 2.3 | Coldness | 1 | 2 | 3 |
| 2.4 | Wet/ slippery | 1 | 2 | 3 |
| 2.5 | Smoke, dust | 1 | 2 | 3 |
| 2.6 | Noise | 1 | 2 | 3 |
| 2.7 | Darkness, lack of illumination | 1 | 2 | 3 |
| 2.8 | Toxic gas | 1 | 2 | 3 |
| 2.9 | Virus, bacteria | 1 | 2 | 3 |
| 2.10 | Unpleasant smell | 1 | 2 | 3 |
| 2.11 | Hot/ inflammable object | 1 | 2 | 3 |
| 2.12 | Sharp object | 1 | 2 | 3 |
| 2.13 | Mentally threatened by others | 1 | 2 | 3 |
| 2.14 | Physically threatened by others | 1 | 2 | 3 |

**3. PHYSICAL FUNCTIONING**

| 3.  Does your current health condition affect any of these following daily functions/ activities? | | | | | Affect a lot | Affect a little | | No effect | |
| --- | --- | --- | --- | --- | --- | --- | --- | --- | --- |
| a | Vigorous physical activities such as lifting heavy object, jogging | | | | 1 | 2 | | 3 | |
| b | Moderate physical activities such as gardening, walking, light exercise | | | | 1 | 2 | | 3 | |
| c | Lifting or carrying a bag (to work, to the supermarket, go out…) | | | | 1 | 2 | | 3 | |
| d | Climbing up several steps | | | | 1 | 2 | | 3 | |
| e | Climbing up one step | | | | 1 | 2 | | 3 | |
| f | Bending your body, kneeling, or bending over | | | | 1 | 2 | | 3 | |
| g | Walking for mother than 1.5 kilometres | | | | 1 | 2 | | 3 | |
| h | Walking for hundred meters | | | | 1 | 2 | | 3 | |
| i | Walking for one hundred meters | | | | 1 | 2 | | 3 | |
| j | Bathing or changing clothes by yourself | | | | 1 | 2 | | 3 | |
| **4. PSYCHOLOGICAL STRESS**  Please listen to each statement and select a number 0, 1, 2 or 3 which indicates how much the statement applied to you over the past week. There are no right or wrong answers. Do not spend too much time on any statement.  0 Did not apply to me at all  1 Applied to me to some degree, or some of the time  2 Applied to me to a considerable degree or a good part of time  3 Applied to me very much or most of the time  **In the past 7 days….** | | | | | | | | |  |
| S1 | | I found it hard to wind down | 0 | 1 | 2 | | 3 | |  |
| S2 | | I tended to over-react to situations | 0 | 1 | 2 | | 3 | |  |
| S3 | | I felt that I was using a lot of nervous energy | 0 | 1 | 2 | | 3 | |  |
| S4 | | I found myself getting agitated | 0 | 1 | 2 | | 3 | |  |
| S5 | | I found it difficult to relax | 0 | 1 | 2 | | 3 | |  |
| S6 | | I was intolerant of anything that kept me from getting on with what I  was doing | 0 | 1 | 2 | | 3 | |  |
| S7 | | I felt that I was rather touchy | 0 | 1 | 2 | | 3 | |  |

**CĂNG THẲNG VÀ CÁC YẾU TỐ LIÊN QUAN Ở CÔNG NHÂN THU GOM RÁC THẢI RẮN ĐÔ THỊ TẠI HÀ NỘI NĂM 2017**

Phỏng vấn viên: ………………………………………………………….......

Ngày phỏng vấn: ………………………………………………………….

Nơi phỏng vấn: …….……………………… Điện thoại: …………………..................

**GIỚI THIỆU**

*Xin chào anh/chị, tôi là……………., ở trường Đại học Y tế công cộng. Hiện nay tôi đang thực hiện một nghiên cứu để tìm hiểu mối liên quan giữa các yếu tố cá nhân và môi trường, kiều kiện làm việc và tình trạng căng thẳng lâm lý ở công nhân thu gom rác. Nghiên cứu này nhằm mục đích cung cấp thông tin nhận diện và đánh giá tình trạng căng thẳng tâm lý mà công nhân thu gom rác có thể gặp phải tại Hà Nội. Kết quả của nghiên cứu góp phần xây dựng các giải pháp phòng chống căng thẳng tâm lý. Toàn bộ thông tin mà anh/chị cung cấp sẽ được đảm bảo bí mật và chỉ dung cho mục đích nghiên cứu. Chúng tôi kỳ vọng anh/chị sẽ tham gia nghiên cứu. Trong quá trình phỏng vấn, anh/chị có quyền từ chối trả lời bất kỳ câu hỏi nào anh chị không muốn trả lời hoặc dừng phỏng vấn bất kỳ khi nào anh/chị muốn.*

*Sau buổi phỏng vấn, nếu anh/chị có bất kỳ câu hỏi nào liên quan đến nghiên cứu, anh/chị có thể liên hệ với chúng tôi qua điện thoại.*

Cuộc phỏng vấn sẽ kéo dài khoảng 20 phút

**Anh/chị có đồng ý tham gia phỏng vấn không?**

[ ] Đồng ý [ ] Từ chối

***Cám ơn sự hợp tác của anh/chị!***

**1. THÔNG TIN CHUNG CHO ĐỐI TƯỢNG NGHIÊN CỨU**

1.1. Năm sinh:…………….

1.2. Giới tính: 1. Nam 2. Nữ 3. Khác

1.4. Trình độ học vấn:

1. Tiểu học 2. THCS 3. PTTH 4. Sơ/ trung cấp 5. Cao đẳng trở lên

1.5. Tình trạng hôn nhân:

1. Chưa kết hôn 2. Kết hôn và sống với vợ/chồng 3. Ly hôn, ly than 4. Khác

1.5. Số con dưới 5 tuổi:

1. Chưa có con 2. Con trên 5 tuổi 3. 1-2 con dưới 5 tuổi 4. > 2 con dưới 5 tuổi

1.6. Anh/chị làm nghề hiện tại được bao nhiêu tháng/năm?........... năm……. Tháng

1.7. Trình bình anh/chị làm việc bao nhiêu giờ/ ngày? …………. Giờ

1.8. Trong 3 tháng qua, anh/chị được phân công làm ca nào?

1. Ca sáng (5h-12h) 2. Ca chiều (1h-8h) 3. Ca tối (6h-2h) 4. Thường xuyên thay đổi

1.8. Thu nhập bình quân hang tháng của anh/chị là bao nhiêu? ……………. Triệu VNĐ

**2. ĐIỀU KIỆN LÀM VIỆC**

Môi trường làm việc của Anh/Chị có các yếu tố nào sau đây?

| **STT** | **Điều kiện làm việc** | **Mức độ tiếp xúc** | | |
| --- | --- | --- | --- | --- |
|  |  | Ko | Ít/ theo mùa | Nhiều |
| 2.1 | Nóng | 1 | 2 | 3 |
| 2.2 | Nắng | 1 | 2 | 3 |
| 2.3 | Lạnh | 1 | 2 | 3 |
| 2.4 | Điều kiện ẩm ướt | 1 | 2 | 3 |
| 2.5 | Khói, bụi | 1 | 2 | 3 |
| 2.6 | Tiếng ồn | 1 | 2 | 3 |
| 2.7 | Tối, không đủ sáng | 1 | 2 | 3 |
| 2.8 | Hơi khí độc | 1 | 2 | 3 |
| 2.9 | Vi trùng, vi khuẩn | 1 | 2 | 3 |
| 2.10 | Mùi hôi thối, khó chịu | 1 | 2 | 3 |
| 2.11 | Chất dễ cháy nổ, bỏng | 1 | 2 | 3 |
| 2.12 | Vật sắc nhọn | 1 | 2 | 3 |
| 2.13 | Bị người khác đe dọa tinh thần | 1 | 2 | 3 |
| 2.14 | Bị người khác đe dọa thể chất | 1 | 2 | 3 |

**3. SỨC KHỎE THỂ CHẤT**

| 5.  Điều kiện sức khỏe hiện nay của anh/chị có làm hạn chế các hoạt động sinh hoạt hàng ngày sau đây? | | | | | Hạn chế rất nhiều | Hạn chế một phần | | Không hạn chế | |
| --- | --- | --- | --- | --- | --- | --- | --- | --- | --- |
| a | Những hoạt động mạnh, ví dụ như nâng vật nặng, chạy bộ… | | | | 1 | 2 | | 3 | |
| b | Những hoạt động vừa phải, ví dụ làm vườn, đi bộ tập thể dục | | | | 1 | 2 | | 3 | |
| c | Nâng hay mang túi đồ (đi làm, đi chợ, đi chơi...) | | | | 1 | 2 | | 3 | |
| d | Leo lên được vài bậc cầu thang gác | | | | 1 | 2 | | 3 | |
| e | Leo lên được một bậc thang gác | | | | 1 | 2 | | 3 | |
| f | Cúi gập người, quỳ xuống hay cúi xuống | | | | 1 | 2 | | 3 | |
| g | Đi bộ hơn 1,5 km (một cây số rưỡi) | | | | 1 | 2 | | 3 | |
| h | Đi bộ vài trăm mét | | | | 1 | 2 | | 3 | |
| i | Đi bộ một trăm mét | | | | 1 | 2 | | 3 | |
| j | Tự tắm rửa hay thay quần áo một mình | | | | 1 | 2 | | 3 | |
| 4 CĂNG THẲNG TÂM LÝ  Anh/chị hãy nghe các câu sau và lựa chọn **01 ý đúng nhất** với anh/chị bằng cách **chọn một trong các số** 0, 1, 2 và 3 ứng với tình trạng mà anh/chị cảm thấy trong suốt **một tuần qua**. Không có câu trả lời đúng hay sai. Và **đừng dừng lại quá lâu ở bất kỳ câu nào**.  0 = Hoàn toàn không đúng  1= Đúng phần nào, thỉnh thoảng  2= Tương đối đúng, nhiều lần  3 = Hoàn toàn đúng  **Trong 1 tuần qua…** | | | | | | | | |  |
| S1 | | Tôi thấy khó mà thoải mái được | 0 | 1 | 2 | | 3 | |  |
| S2 | | Tôi có xu hướng phản ứng quá mức với mọi tình huống | 0 | 1 | 2 | | 3 | |  |
| S3 | | Tôi thấy mình đang suy nghĩ quá nhiều | 0 | 1 | 2 | | 3 | |  |
| S4 | | Tôi thấy bản thân dễ bị kích động | 0 | 1 | 2 | | 3 | |  |
| S5 | | Tôi thấy khó mà thư giãn được | 0 | 1 | 2 | | 3 | |  |
| S6 | | Tôi không chấp nhận được việc có cái gì đó xen vào cản trở việc tôi đang làm | 0 | 1 | 2 | | 3 | |  |
| S7 | | Tôi thấy mình dễ bị phật ý, tự ái | 0 | 1 | 2 | | 3 | |  |
